# Soft, breathable, and recyclable MXene fabrics for wearable electrophysiological recordings†

**DOI:** 10.1039/d5mh00831j

**Published:** 2025-07-04

**Authors:** Kihyun Lee, Osvaldo Linares Gutierrez, Wubin Bai

**Affiliations:** a Department of Applied Physical Science, University of North Carolina at Chapel Hill Chapel Hill North Carolina 27514 USA wbai@unc.edu

## Abstract

The widespread utility of single-use bioelectronic devices, particularly disposable electrodes for electrophysiological monitoring, raises environmental concerns due to increased medical waste and non-biodegradable materials. This underscores the growing demand for wearable electrode systems that deliver high signal fidelity while adhering to sustainability principles. This study presents a recyclable, wearable electrode patch incorporating a gelatin matrix embedded in Ti_3_C_2_T_*x*_ MXene non-woven fibers manufactured *via* wet spinning. This design enables repeated reprocessing at low temperatures, around 40 °C, due to the thermoreversible solution–gel properties of gelatin, allowing for multiple cycles of reuse without performance degradation. The MXene/gelatin non-woven structure maintains high conductivity, mechanical flexibility, and skin compatibility while exhibiting excellent breathability. The fine fiber structure and controlled deposition provide enhanced interfacial electrical conductivity and adjustable density depending on fiber diameter. As a result, the manufactured non-woven fabric electrode demonstrates low impedance, a high signal-to-noise ratio, and reliable acquisition of bio-signals from skin. Electrocardiogram and electromyogram measurements showed stable performance even after recycling, proving the potential of conventional electrodes as an alternative. This study presents an integrated approach that achieves both functional performance and environmental sustainability in eco-friendly bioelectronics.

## Abstract 2

Recyclable, breathable MXene/gelatin non-woven fabric electrodes for sustainable, reliable, and stable electrophysiological recordings.

## Graphical abstract

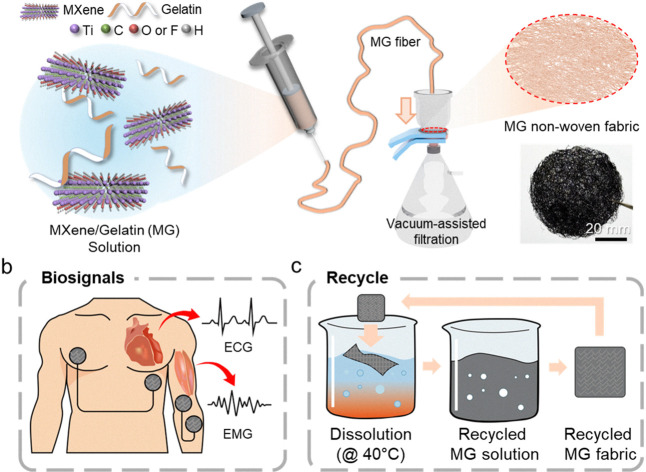

**New concepts**

Currently, wearable bioelectronic sensors are widely used for continuous physiological monitoring but are designed as single-use devices. As a result, they contribute significantly to biomedical waste and environmental burden, mainly due to the incorporation of synthetic polymers, non-degradable substrates, and metal-based components. In addition, wet gel electrodes may have a limited lifespan due to gel dehydration and cause skin irritation. Dry electrodes can improve comfort, but they have limitations such as high impedance and reduced signal stability during activity. These functional and environmental limitations remain major obstacles to the long-term application of biosensors. To address this, we introduce a recyclable, air-permeable skin-interfacing electrode composed of a nonwoven fabric made from a Ti_3_C_2_T_*x*_ MXene and gelatin composite. The thermoreversible behavior of gelatin enables reshaping and multi-cycle reuse through mild heating (∼40 °C), avoiding material waste and preserving signal quality. The nonwoven network of microscale entangled fibers ensures excellent breathability, conformal contact, and stable electrical performance across hydration states. This synergistic material platform allows reliable ECG and EMG signal acquisition while meeting sustainability, biocompatibility, and wearability demands. Our work offers a pathway toward environmentally responsible and functionally resilient wearable bioelectronics for next-generation medical and personal health applications.

Monitoring bioelectric signals (*e.g.*, electrocardiograms (ECG) and electromyograms (EMG)) continuously and non-invasively at the skin interface is essential for early diagnosis, rehabilitation, and personalized medical services.^1–5^ In recent years, the widespread use of disposable skin contact sensors such as ECG and EMG electrodes has significantly contributed to the increase in medical-related biological waste.^6–8^ Approximately 313 million surgeries are performed annually, mostly involving multiple disposable sensors.^6,7^ This trend has been further accelerated by the COVID-19 pandemic, which has led to stricter infection control standards and a surge in demand for disposable medical devices.^9,10^ In addition, concerns about hygiene and device performance degradation mean that these skin contact electrodes are generally not reused.^11^ As a result, they are regularly disposed of after single use, contributing significantly to the medical waste stream. U.S. healthcare facilities generate approximately 14 000 tons of waste daily, with a significant portion coming from single-use medical supplies, including electrodes and wearable sensors.^12^ The environmental impact of traditional electrode materials further exacerbates these concerns.^13^

Currently, widely used skin electrodes are typically composed of silver/silver chloride (Ag/AgCl) patches coated with a moisture-retaining gel or dry electrodes made from nano-scale or highly porous materials.^14–17^ These electrodes have been adopted as clinical standards due to their low resistance and high signal fidelity. However, gel-based electrodes have inherent limitations, including a limited service life due to gel dehydration and skin irritation from prolonged use.^18,19^ In addition, Ag/AgCl electrodes used in conjunction with them may release environmental residues and toxic silver ions if mishandled, causing harmful effects on the environment.^20–22^ Additionally, the hydrogel used in gel-based electrodes is composed of synthetic polymers such as polyacrylamide or polyvinyl alcohol, which have low biodegradability and can accumulate in ecosystems.^23–25^ Dry electrodes offer improvements over gel-based electrodes in terms of long-term comfort, mechanical durability, and partial environmental friendliness.^26–28^ However, they also have drawbacks. Direct skin contact without an intermediate moisture-regulating gel layer can lead to moisture regulation and mechanical mobility issues, reducing data consistency and reliability during continuous or motion-intensive monitoring.^29^ In addition, dry electrode systems are often manufactured using materials that are difficult to biodegrade, which can compromise overall environmental sustainability.^30^ Therefore, developing bioelectronic interfaces that satisfy both performance and environmental considerations is becoming increasingly important.

Recent advancements in the field of two-dimensional materials have opened new avenues for the development of high-performance wearable electrodes. MXenes, a class of two-dimensional transition metal carbides and nitrides including Ti_3_C_2_T_*x*_, exhibit excellent properties such as high electrical conductivity (>20 000 S cm^−1^), hydrophilicity, mechanical flexibility, and ease of solution processing.^31–34^ However, simple planar MXene films lack breathability and are prone to mechanical delamination under repeated deformation, particularly when adhered to dynamic skin surfaces.^35,36^ In addition, these films often exhibit limited structural flexibility due to their continuous and dense morphology, making them less suitable for conformal contact during motion. To overcome these limitations, switching from film to fiber-based structures is gaining some attention.^37,38^ Fiber materials are naturally flexible and thin, making them breathable and able to adapt to changes in shape.^39–42^ However, single fibers alone cannot provide mechanical integrity or robust signal transmission for epidermal applications.^43^ When these fibers are assembled into a non-woven network, the resulting inter-fiber entanglement enables distributed load sharing and enhanced structural stability, reducing localized mechanical strain and improving contact robustness.^44^ This network structure can adapt better to dynamic movements while maintaining electrical performance, making it particularly advantageous for long-term, motion-resistant acquisition of biological signals.^44^ Therefore, a non-woven MXene fiber network holds substantial potential as a dry, breathable, and flexible platform for high-performance wearable electrodes such as those used in ECG and EMG monitoring systems.

Gelatin is a naturally derived biopolymer obtained through the partial hydrolysis of collagen, possessing unique properties that make it highly suitable for biomedical applications.^45^ It inherently exhibits biocompatibility, biodegradability, water solubility, a relatively low melting point (∼35–40 °C), and thermoreversible solution–gel transition characteristics.^46,47^ These properties enable gelatin-based materials to undergo mild thermal processing and reformation without chemical degradation. Additionally, gelatin's soft and hydrophilic nature promotes close contact with biological tissues, reduces mechanical mismatch at interfaces, minimizes stimulation during repeated deformation, and enhances device stability.^48^

In this study, we combined MXene fibers manufactured *via* a wet spinning process with a gelatin matrix to develop a recyclable dry electrode platform. A non-woven structure confers high porosity, mechanical flexibility, and adaptability to dynamic skin surfaces while ensuring a robust planar external electrical pathway essential for stable signal acquisition. With a composition of interwoven microfibers, non-woven constructs naturally offer high porosity, mechanical strength, excellent breathability, and excellent conformity to complex and changing skin surfaces. Fiber entanglement is key to the uniform distribution of mechanical stress and, hence, the resistance to stretching, compression, and bending. Additionally, reducing the fiber diameter exponentially improves vertical conductivity (*σ* ∝ *D*^−4^), directly enhancing the efficiency of bio-signal acquisition. The composite with gelatin enables thermal reprocessing under mild conditions (∼40 °C), allowing for multiple cycles of reuse and reconfiguration without performance degradation. The functional effectiveness of the MXene/gelatin non-woven electrode was validated by recording high-quality ECG and EMG signals with low motion artifacts and low skin-electrode impedance. The electrode showed an improved signal-to-noise ratio under resting and dynamic states, thus confirming its mechanical and electrical stability under real-life wearable conditions. This study demonstrates the potential of disposable bioelectronic interfaces as an environmentally friendly alternative, showing great potential for contributing to developing next-generation wearable medical devices that consider both performance and sustainability.

## Results and discussion

### Fabrication and conceptual overview of the MXene/gelatin non-woven fabric

**Fig. 1:**
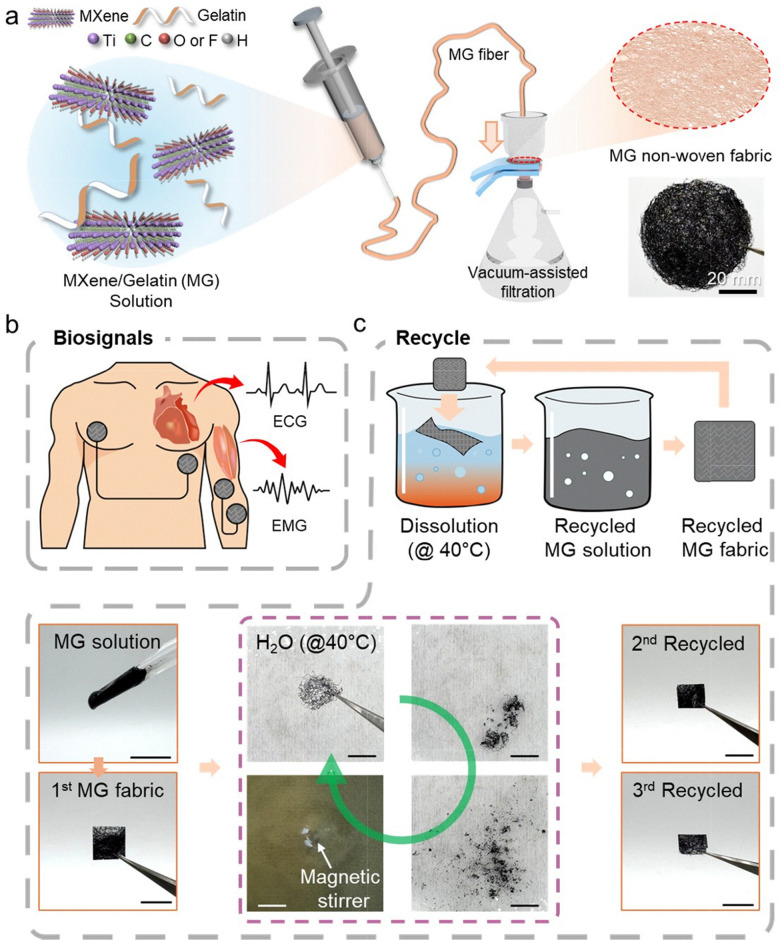
Schematic illustration of the overall process and application of the MXene/gelatin (MG)-based non-woven fabric. (a) A conceptual illustration and photo image of the wet spinning of the MG solution and the entanglement process by vacuum-assisted filtration. (b) Wearable application of the fabric for biosignal monitoring, including ECG and EMG measurements. (c) A schematic illustration and a series of images showing the process of dissolution and 3 times of recycling of the fabric electrode during immersion in deionized water at a temperature of about 40 °C. Scale bar, 1 cm.

The overall manufacturing process and application concept of MXene/gelatin (MG) non-woven fibers are schematically illustrated in Fig. 1. In this study, Ti_3_C_2_T_*x*_ MXene nanosheets were uniformly dispersed in a gelatin and aqueous solution. The MXene solution was prepared at a concentration of 28 mg mL^−1^ using a modified etching method,^49,50^ and internal bubbles were removed through continuous stirring and a short ultrasonic dispersion process. Each MXene and gelatin were prepared as solutions and confirmed to have a single layer with an average thickness of approximately 3 nm and surface functionalities (Fig. S1–S3, ESI†). Additionally, gelatin was first prepared at a concentration of 2 wt%, and MXene solution was added at various volume ratios to prepare composite solutions. Subsequently, continuous MG fibers were produced *via* a controlled wet spinning process (Fig. 1a), where the fibers solidified in a coagulation bath. The produced fibers were manufactured into an entangled, porous, and independent non-woven fiber structure *via* vacuum-assisted filtering. This strategy realized a textile structure with flexibility, mechanical strength, and high conductivity optimized for skin-adaptive bioelectronic applications. As shown in Fig. 1b, the MG non-woven fabric can be used as a wearable electrode in direct contact with the skin surface to measure bioelectric signals such as electrocardiogram (ECG) and electromyogram (EMG) signals. In addition, the introduction of gelatin provides thermoreversible properties even at low temperatures of ∼40 °C, enabling nonwoven fibers to dissolve and reform in water (Fig. 1c). This plays an important role to realize sustainability in the wearable electronic devices.

### Analysis of the structural and electrical properties

**Fig. 2:**
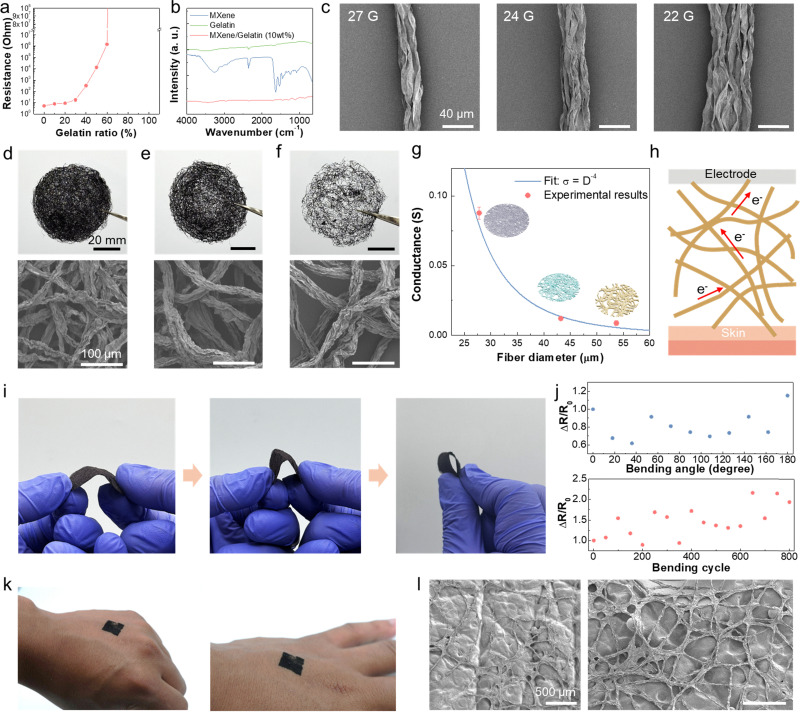
Morphological and electrical characterization of the MXene/gelatin (MG) nonwoven fabric. (a) Resistance variation of MXene/gelatin composites as a function of gelatin content, demonstrating the effect of gelatin ratio on electrical properties. (b) Fourier-transform infrared (FT-IR) spectra of pure MXene, pure gelatin, and the MXene/gelatin (10 wt%) composite, confirming chemical interactions between components. (c) Scanning electron microscopy (SEM) images of individual MG fibers fabricated using needles with different diameters. (d)–(f) Photo images of the overall nonwoven fabric structure prepared using different needle gauges (22 G, 24 G, and 27 G) with the same volume (1 mL) of MG solution, along with magnified SEM views highlighting the detailed microstructure of the fibers and their packing density. (g) Graph illustrating the relationship between out-of-plane resistance and fiber diameter, showing an inverse fourth-power dependence (*σ*_⊥_ ∝ *D*^−4^). (h) Schematic illustration of out-of-plane charge transport pathways within the fiber network, with an inset depicting how fabric density varies with needle diameter through changes in inter-fiber contact points. (i) Optical image of the MG nonwoven fabric undergoing mechanical bending. (j) Demonstration of mechanical robustness and flexibility of the freestanding nonwoven fabric under various bending angles, accompanied by resistance measurements during cyclic bending tests. (k) Photograph of the MG nonwoven fabric conformally attached onto the human skin, demonstrating intimate skin contact without delamination. (l) SEM image of the MG nonwoven fabric adhered to a skin-mimicking replica, showing conformal adhesion and structural integrity at the skin interface.

First, the resistance of each MXene/gelatin composite was measured by varying the mixing ratio of MXene and gelatin (Fig. 2a). Until 30 wt% gelatin was mixed, the resistance of pristine MXene remained relatively stable, but a rapid increase in resistance was observed beyond this ratio. Therefore, we fixed the gelatin ratio at 10 wt% for further experiments. Fourier-transform infrared spectroscopy was used to identify the characteristic peaks of each material in the solution and structure. In the mixed samples, both MXene and gelatin peaks were clearly observed. This confirms that the mixing of two materials was successful (Fig. 2b). Next, the morphology of MG single fibers produced using various needle diameters was examined using scanning electron microscopy (SEM) (Fig. 2c). The non-woven fabrics made by continuous extrusion of MG solutions, where single fibers aggregate, exhibit a more compact structure and clearly defined fiber networks compared to samples made using the conventional method with crushed fibers (Fig. 2d–f).^51,52^ Furthermore, when the same volume of MG solution was used, the fiber diameter and entanglement density varied depending on the needle diameter, demonstrating a denser fiber network. Fibers produced using a smaller needle diameter (27 G; inner diameter (I.D.) is 0.21 mm) exhibited smaller inter-fiber spacing and a more compact structure, while fibers produced using larger needle diameters (22 G; I.D. is 0.413 mm and 24 G; I.D. is 0.311 mm) showed a more loosely packed structure. The diameter differences of individual fibers caused by the needle were confirmed by comparing the calculated values based on the cylindrical structure with the actual out-of-plane conductivity of the non-woven fiber samples using SEM images.

In particular, it was confirmed that the density of the non-woven structure can significantly influence the planar electrical conductivity. Based on the relationship between fiber density and the number of fiber contact points, a theoretical relationship between fiber diameter (*D*) and out-of-plane conductivity (*σ*_⊥_) was derived through a simple proportional equation. In detail, we analyzed the influence of fiber diameter on the structural and electrical properties of non-woven fabrics by considering the number of fibers (*N*) contained within a fixed total volume (*V*). First, each fiber was assumed to have a cylindrical geometry, and its volume is given as follows:
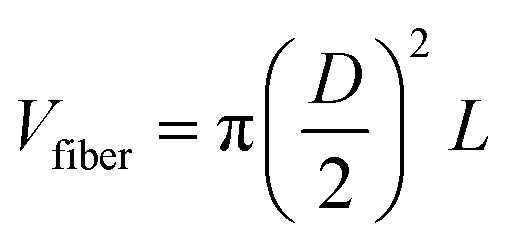
*D* is the fiber diameter, and *L* is the average fiber length. Assuming that the packing fraction *ϕ* (defined as the ratio of the mass or volume fraction of solid content (MXene and gelatin) in the spinning solution) is constant, the total number of fibers *N* is expressed as follows:
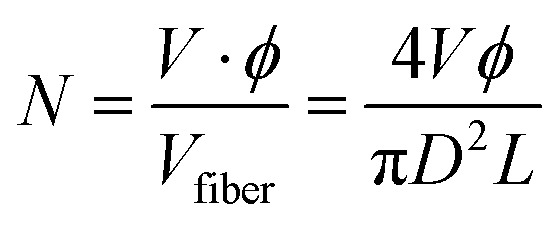
Therefore, the number of fibers is inversely proportional to the square of the fiber diameter (*N* ∝ *D*^−2^). As the fiber diameter decreases, the number of fibers per unit volume increases significantly. The number of contact points *C* within a non-woven fabric composed of manufactured fibers is critical for forming efficient charge transfer pathways. Statistically, if the potential number of contacts is proportional to the number of unique fiber pairs, it exhibits a quadratic dependence on *N* (*C* ∝ *α*·*N*^2^). Here, *α* represents the contact probability between fibers, which can be influenced by fiber orientation or surface roughness. Substituting this relationship into *N* yields *C* ∝ (*D*^−2^)^2^ = *D*^−4^. Therefore, as the fiber diameter decreases, the number of contact points between fibers increases exponentially. The out-of-plane electrical conductivity *σ*_⊥_ is directly proportional to the number of available vertical conduction paths, which is proportional to the number of contact points (*σ*_⊥_ = *γ*·*C*·*σ*_contact_). Here, *γ* is a geometric factor of vertical current paths, and *σ*_contact_ denotes the intrinsic conductivity of individual fiber–fiber junctions. Combining these relationships, the out-of-plane conductivity scales as follows: *σ*_⊥_ ∝ *C* ∝ *D*^−4^. As the fiber diameter decreases, the macroscopic out-of-plane electrical conductivity of non-woven materials increases exponentially. For example, doubling the fiber diameter theoretically increases the planar-out-of-plane electrical conductivity by a factor of 16, emphasizing the importance of optimizing the fiber microstructure in the design of high-performance bioelectronic materials. Fig. 2g shows the out-of-plane electrical conductivity of non-woven fabrics made with various needles as a function of fiber diameter, confirming the scaling trend inversely proportional to the fourth power of the fiber diameter, as mentioned earlier, and indicating that conductivity decreases exponentially as the fiber diameter increases. In Fig. S4 (ESI†), 2.5 mL and 2.0 mL of MG solution were used when 22 G and 24 G needles were used to achieve electrical conductivity similar to that of 27 G fibers. Considering the theoretical relationship, the 22 G needle requires approximately 13.92 times more solution, and the 24 G needle requires approximately 5.83 times more. However, under fixed area conditions, increasing the number of fibers forms a more densely packed and entangled network, resulting in more actual contact points than predicted by the ideal model. This can explain why the out-of-plane electrical conductivity of the larger number of 22 G and 24 G samples (2.5 mL and 2 mL, respectively) exceeded the theoretical expectations (Fig. S4, ESI†). However, this was insufficient to offset the drawbacks of reduced flexibility and decreased manufacturing efficiency relative to solution usage, as the thickness of the fabric electrode increased proportionally to the amount of solution used. These results clearly demonstrate that it is essential to optimize the microfiber structure to improve the charge transfer path in the mechanism by which the nonwoven electrode obtains signals (Fig. 2h).

The mechanical strength and flexibility of the MG nonwoven fabric were evaluated by bending tests in which the fabric was repeatedly bent at various angles (Fig. 2i). The fabrics were exposed to dynamic mechanical deformations, including sharp bending and folding, to mimic the mechanical stresses encountered in skin-adhesive wearable devices. The results show that the non-woven fabric maintained its fiber network structure and electrical conductivity even after more than 600 bending cycles. Electrical continuity was simultaneously monitored during mechanical testing. As shown in Fig. 2j, resistance measurements collected during repeated bending exhibited only minor fluctuations in resistance values, indicating negligible deviations even under repeated high-deformation bending. This consistency highlights the excellent mechanical durability of MG non-woven fabrics, which can be attributed to the highly intertwined porous fiber network effectively distributing mechanical stress across multiple fibers and contact areas. Repetitive bending over 2000 cycles leads to an apparent increase in resistance values, along with the propagation of cracks within the fiber structure, suggesting cumulative mechanical fatigue and the gradual degradation of electrical pathways (Fig. S5, ESI†). Nevertheless, the overall performance remained stable for a significant number of cycles, demonstrating excellent fatigue resistance of our electrodes for wearable applications. MXene/gelatin composites exhibit enhanced electrical stability and delayed structural degradation compared to pure MXene during repeated wear cycles (Fig. S6, ESI†). Unlike thin film electrodes, the three-dimensional intertwined structure of MG non-woven materials provides inherent deformation mitigation pathways that prevent rupture or damage even under repeated loading.

MG materials attached to actual human skin (Fig. 2k) and skin-mimicking replicas with complex curvature and texture (Fig. 2l) demonstrated excellent shape-adaptive adhesion. The non-woven structure adheres closely to the skin's microstructure, preventing separation, wrinkling, or peeling, which is essential for stable biological signal acquisition, noise minimization, and low impedance results. In addition, to simulate real-world situations, planar conductance was measured under varying normal forces (0–2.0 N), revealing that increased pressure improved contact quality by enhancing interface conformity, particularly in electrodes with rough surfaces (Fig. S7, ESI†). The synergistic combination of mechanical strength, flexibility, and adaptability demonstrated here is essential for practical wearable bioelectronic applications. Electrodes placed on dynamic and irregular surfaces like the human body must withstand static deformation and continuous mechanical stress over extended periods. The electrical and structural performance stability demonstrated by MG non-woven fibers under these conditions highlights their potential as a reliable and durable platform for long-term physiological monitoring.

### Evaluation of breathability, moisture permeability, and recyclability

**Fig. 3:**
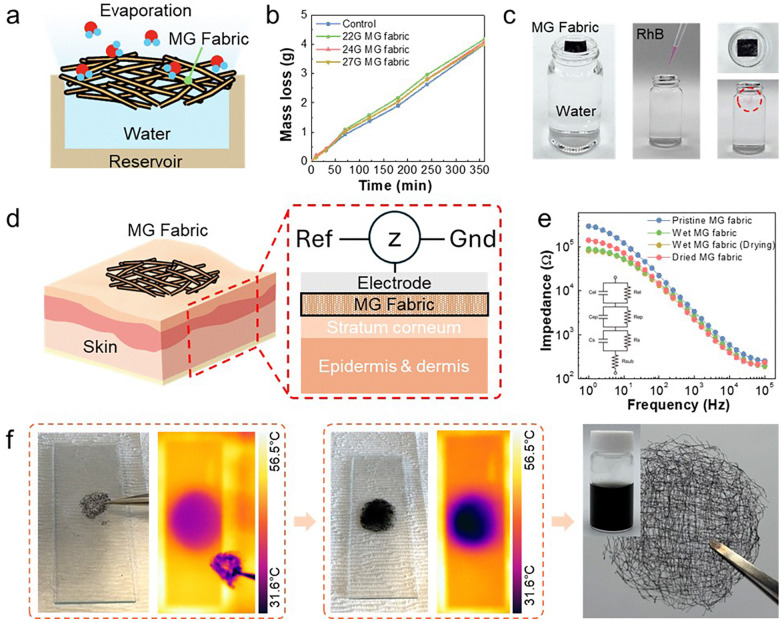
Evaluation of breathability, permeability, and recyclability of the MG non-woven fabric. (a) Schematic illustration of the water evaporation test setup using a sealed reservoir, designed to evaluate the breathability of the MG non-woven fabric under controlled conditions. (b) Time-dependent water evaporation rates measured through MG fabrics fabricated with different fiber densities, demonstrating the influence of structure on moisture permeability. (c) Dye diffusion test showing the permeability of the MG fabric, where the penetration of a dye solution through the fabric into the underlying water reservoir was monitored. (d) Schematic diagram of the experimental setup for skin impedance measurement, including a structural model for the electrode–skin interface. (e) Photographs demonstrating the recyclability of the MG non-woven fabric: dissolution of the fabric in 40 °C water, recovery of the MG solution, and reformation of a new non-woven fabric from the recycled material. (f) Impedance spectra of the MG fabric recorded at different drying stages (immediately after wetting, after 30 minutes of drying, and after complete drying) between the fabric and the skin.

The breathability and moisture permeability of MG nonwoven fibers were studied to evaluate their suitability for long-term epidermal application. Physiologically, human skin experiences moisture absorption due to sweat and continuous moisture loss, so ensuring sufficient moisture permeability is essential for skin health and wear comfort in wearable bioelectronic devices.^53,54^ To quantitatively evaluate vapor permeability, an experimental setup mimicking physiological evaporation processes was designed (Fig. 3a).^55,56^ Under controlled environmental conditions (approximately 38 °C, relative humidity 45–50%), weight loss due to vapor evaporation was recorded over time in a water reservoir sealed with the MG non-woven material. For comparison, a traditional filter membrane (0.45 mm pore size) was also tested under the same conditions. As shown in Fig. 3b, the MG non-woven material demonstrated a high water vapor transmission rate (WVTR) similar to that of the traditional filter membrane, indicating minimal obstruction to moisture release. Specifically, the WVTR of the high-density (27 G) MG fabric was measured at approximately 68.55 mg cm^−2^ h^−1^, demonstrating superior permeability compared to the traditional filter film (67.58 mg cm^−2^ h^−1^). Low-density MG fabrics (22 G and 24 G) also exhibited high WVTR values of 71.33 and 68.64 mg cm^−2^ h^−1^, respectively. This is because low-density fabrics ultimately contain larger structural pores. The WVTR values of two commercial patches, Hypafix and Tegaderm, were found to be 5.60 and 0.62 mg cm^−2^ h^−1^, respectively, for comparison (Fig. S8, ESI†). Our patch exhibited a higher WVTR than both of its commercial counterparts. Therefore, it can be concluded that high-density non-woven fabric structures also exhibit excellent permeability, which is attributed to the inherent porosity and hydrophilicity of the non-woven structure, as well as the fiber network structure that promotes water vapor movement.

A dye permeability test was conducted to evaluate permeability further. A droplet of an aqueous Rhodamine B dye solution (20 μL) was carefully applied to the surface of the MG fabric, and the time required for the dye to pass through the fabric and appear on the lower absorbent layer was recorded (Fig. 3c). The MG fabric demonstrated an initial permeability time of less than 1 second, indicating excellent permeability performance. The sustained permeability performance of the MG fabric promotes uniform evaporation of moisture and prevents localized moisture accumulation, thereby supporting practical moisture management functionality that maintains skin comfort during prolonged wear.

Impedance measurements were performed to evaluate the electrochemical properties of the non-woven fabric when in contact with the skin (Fig. 3d).^57,58^ The impedance spectra recorded for the MG fabric and those recorded immediately after wetting and after drying are shown in Fig. 3e. An equivalent circuit model was additionally used to analyze the skin–fabric interface impedance characteristics. The MG non-woven material maintained a low impedance even when completely dried. Furthermore, when moisture was present, our non-woven fabric drying electrodes exhibited enhanced contact with the skin, resulting in lower impedance. These results confirm that the MG non-woven fabric remains mechanically, structurally, and electrically stable after repeated wetting and drying cycles, even when exposed to water for extended periods, and maintains adhesion to the skin model. Overall, these results demonstrate that MG non-woven materials possess excellent breathability, moisture permeability, and moisture resistance, positioning them as ideal candidates for long-term high-performance epidermal bioelectronic devices. While most traditional electrodes suffer from poor skin compatibility due to insufficient permeability, MG non-woven materials successfully combine electrical functionality with superior physiological comfort.

The recyclability of MG non-woven materials has been systematically demonstrated through a mild thermal process, emphasizing the sustainability and reusability of the material system. As shown in Fig. 3f, the fabricated MG non-woven material was introduced into a controlled dissolution process in deionized water maintained at ∼40 °C. This temperature was carefully selected to maintain the integrity of MXene nanosheets while efficiently promoting gelatin dissolution. After immersion, the material gradually decomposed as the gelatin matrix underwent a thermodynamically reversible sol–gel transition. Gelatin, a partially denatured form of collagen, exhibits a low critical dissolution temperature (∼35–40 °C) in an aqueous environment, allowing polymer chains to regain mobility and solubility. This mild thermal activation enabled rapid dispersion of gelatin and MXene components into a uniform solution through mechanical dispersion alone, without harsh chemicals, thereby minimizing potential damage to the conductive MXene structure.

After complete dissolution, the resulting MG aqueous solution was re-introduced for wet spinning. The solution was loaded into a syringe and extruded through a spinneret under controlled conditions and then a coagulation bath to form regenerated MG fibers. The newly formed fibers were reconstituted into a non-woven fiber structure through vacuum-assisted entanglement and filtering. In particular, the regenerated MG fibers exhibited mechanical and electrical properties similar to those of the initial MG fabric. This closed-loop reprocessing capability reduces material consumption and environmental impact and enables on-site customized production and repair.

### Biological signal monitoring applications

**Fig. 4:**
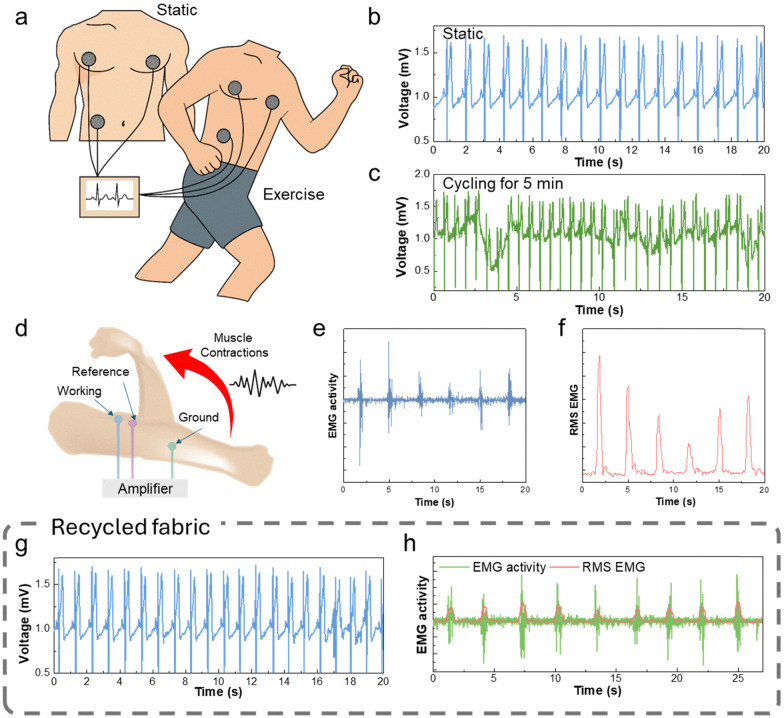
Application of the nonwoven fabric as a biosignal monitoring electrode. (a) Schematic illustration of the ECG measurement setup with electrode placement on the human upper body and photo image of the non-MG woven fabric electrode. (b) ECG signal recorded using the nonwoven MG fabric electrode. (c) ECG signals recorded under exercise from our MG fabric during 5 min of cycling. (d) 2D schematic illustration of the EMG measurement setup showing electrode placement and targeted muscle movement in the arm. (e) Recorded EMG signals during muscle activity. (f) Root mean square (RMS) values of EMG signals for samples with different conductivities. (g and h) The ECG (g) and EMG (h) performances after recycling of the MG non-woven fabric.

MG non-woven fabrics' biological signal monitoring performance was comprehensively evaluated through ECG and EMG recordings (Fig. 4a). To assess the excellent biological signal monitoring capability of MG non-woven materials, ECG and EMG signals were recorded using electrodes fabricated under 27 G needle conditions and evaluated comprehensively. The fine fiber diameter (∼27.8 μm) and high density achieved by this specification enhance planar conductivity and enable close contact with the skin, contributing to reliable signal acquisition. Continuous ECG recordings were performed during measurement cycles conducted through rest and mild exercise stages. ECG signals under sweat exposure maintained consistent quality, with detailed waveforms confirmed in the magnified view (Fig. S9, ESI†). Clear and stable waveforms were obtained during the measurement period, with distinguishable P waves, sharp QRS complexes, and T wave recovery observed.^59^ Notably, the signal morphology remained consistent even under mild movement, demonstrating the excellent mechanical adaptability of the non-woven material interface. Signal quality was quantified using root mean square (RMS) analysis and signal-to-noise ratio (SNR) metrics. The RMS-based SNR of the ECG signal was calculated to be approximately 34.28 dB, reflecting an appropriate yet sufficient separation between baseline noise and cardiac signal amplitude. Fig. S10 (ESI†) shows that stable ECG signals were recorded for a total of 10 hours, with 2 hours of monitoring per day for five consecutive days, demonstrating the device's long-term monitoring capability.

MG electrodes were placed on the biceps brachii muscle group to perform EMG measurements, and muscle contraction signals were recorded at 3-second intervals during target movements (Fig. 4d). A clear surge in electrical activity corresponding to voluntary muscle activation was observed (Fig. 4e), demonstrating the sensitivity and responsiveness of the MG electrodes to physiological signals.^60^ Using RMS analysis (Fig. 4f), quantitative evaluation of EMG signals revealed a positive correlation between fabric conductivity and EMG signal amplitude. RMS-based EMG data analysis yielded an SNR of approximately 15.2 dB, indicating distinguishable signal amplitude relative to baseline noise during voluntary muscle contraction. EMG signals also remained stable even after 5 days of electrode use, demonstrating the durability and long-term performance of the MG non-woven electrode (Fig. S11, ESI†).

Importantly, the recyclability and reusability of the MG non-woven fabric were verified through ECG and EMG measurements after the recycling cycle (Fig. 4g and h). The regenerated electrodes maintained mechanical stability and high conductivity when the original MG fabric was dissolved in 40 °C water and reformed into a new non-woven structure. ECG and EMG signals recorded from the recycled patches exhibited performance levels similar to those of the original pure samples, with little change in signal fidelity, SNR, or baseline stability. The successful recyclability is attributed to several factors. First, the mild reprocessing temperature prevents severe oxidation of MXene particles, which are prone to degradation at high temperatures or in oxidizing environments. Second, the dissolution process in water does not cause severe separation or re-layering of MXene layers due to the stabilizing effect of the gelatin matrix between MXene layers. Gelatin acts as a protective dispersant, minimizing the aggregation of MXene nanosheets and maintaining uniform dispersion even after reprocessing. The reconstituted MG solution also maintains sufficient concentration to minimize structural and electrical property losses during fiber spinning and network reformation processes. XPS analysis after three recycling cycles revealed distinct Ti2p and C1s peaks of MXene, confirming the preserved chemical structure of the fabric (Fig. S12, ESI†). In addition, under 100% relative humidity, the MXene/gelatin fabric exhibited significantly greater resistance stability over time compared to pure MXene, as shown in Fig. S13 (ESI†). These results emphasize the robustness, durability, and sustainability of MG non-woven fibers as dry electrode materials for wearable bioelectronic devices. By maintaining high signal quality even after repeated mechanical deformation, prolonged skin contact, dynamic movement, and multiple recycling cycles, MG fibers have demonstrated their potential as an innovative material platform capable of addressing the key challenges in developing long-term, sustainable, high-performance epidermal electronic systems.

## Conclusions

In this study, we successfully developed a reusable wearable electrode platform based on an MG non-woven fiber structure optimized for wearable biosignal monitoring. By combining the excellent electrical conductivity of Ti_3_C_2_T_*x*_ MXene with the biocompatibility and thermoreversibility of gelatin and manufacturing them into a fiber network-based non-woven fabric structure, we were able to produce fabric-type electrodes with mechanical flexibility, moisture permeability, breathability, and skin adhesion. By strategically designing the fiber microstructure through wet spinning and vacuum-assisted entanglement, we precisely controlled the in-plane conductivity, which exhibits an inverse square relationship (*σ*_⊥_ ∝ *D*^−4^) inversely proportional to the fiber diameter. This emphasizes the importance of fiber network design for optimizing vertical charge transport. In particular, the introduction of gelatin enables recycling without compromising electrode performance even under mild thermal conditions (∼40 °C). This suggests a sustainable research direction for addressing environmental issues associated with disposable bioelectronic electrodes and sensors. The MG non-woven fiber platform proposed in this study is expected to enable the development of fully autonomous, long-lasting wearable devices by integrating it into future research. Furthermore, by controlling the intrinsic properties of MXene, it will be possible to integrate additional sensing technologies such as pH and temperature sensing modalities, demonstrating strong potential for expansion into multifunctional wearable systems.

## Experiments on human subjects

The human subject study was approved by the Office of Human Research Ethics at the University of North Carolina at Chapel Hill (Protocol no. 22-0163). All human subjects gave written and informed consent before participating in the studies.

## Author contributions

Kihyun Lee: conceptualization, data analysis, investigation, methodology, and writing of the original draft; Osvaldo Linarez: data acquisition, analysis, and investigation; and Wubin Bai: conceptualization, resources, project administration, supervision, funding acquisition, and writing – review and editing.

## Conflicts of interest

There are no conflicts to declare.

## Supplementary Material

MH-012-D5MH00831J-s001

## Data Availability

The data supporting the findings of this study are included in the ESI† of this article.
