# Supplementary material for: Soft, breathable, and recyclable MXene fabrics for wearable electrophysiological recordings
Source: Mater Horiz. 2025 Jul 4;12(20):8528–36. doi: 10.1039/d5mh00831j (PMC12282577; doi:10.1039/d5mh00831j)
Supplement: MH-012-D5MH00831J-s001 [file MH-012-D5MH00831J-s001.pdf]

Supplementary Information (SI) for Materials Horizons.
This journal is © The Royal Society of Chemistry 2025

[Supporting Information to accompany manuscript]

Soft, Breathable, and Recyclable MXene Fabrics for Wearable Electrophysiological Recordings

Kihyun Lee, Osvaldo Linares Gutierrez, and Wubin Bai*

Department of Applied Physical Science, University of North Carolina at Chapel Hill, Chapel Hill, North Carolina, 27514, United States

*Correspondence and request for the materials should be addressed to W.B. (wbai@unc.edu)

MATERIALS AND METHODS

S1. Materials

Layered ternary carbide (Ti_3AlC_2) MAX-phase powders, hydrochloric acid (HCl), lithium fluoride (LiF), ammonium chloride (NH_4Cl, ACS reagent, ≥ 99.5%), gelatin (from porcine skin, Type A, gel strength ~300 g Bloom) were purchased from Sigma-Aldrich Co. (USA). Ultrapure deionized (DI) water (>18.2 MΩ cm of resistivity) was obtained from a ACME Analytical Solutions. Standard artificial sweat solution (ISO 3160-2) was purchasead from Fisher Scientific. Polydimethylsiloxane (PDMS; Sylgard 184 silicone elastomer kit) was purchased from Dow Corning. Dragon Skin^{TM} (10 very fast) was obtained from Smooth-On Inc.

S2. Synthesis of the $Ti_3C_2T_x$ MXene

The MXene was synthesized from Ti_3AlC_2 (MAX phase) by modifying an established etching approach.[1,2] Initially, 2 g of lithium fluoride (LiF) was dissolved in 40 mL of 9 M hydrochloric acid (HCl) in a three-neck round-bottom flask. The mixture was stirred at 35 °C for 30 minutes to ensure complete dissolution. MAX phase powder (2 g) was then gradually introduced to the solution under continuous stirring in an argon atmosphere, and the etching process was carried out for 24 hours. This treatment selectively removed the aluminum layer from the MAX phase, resulting in the exfoliation of $Ti_3C_2T_x$ MXene layers. Post-etching, the reaction mixture was aliquoted into 20 mL conical tubes and further diluted with deionized water in a 1:1 ratio. The dispersion was repeatedly centrifuged and washed with DI water to eliminate residual acid, until the pH stabilized around 6. As the pH increased, the negative surface charge of the MXene flakes was enhanced, which led to their stable suspension and resistance to sedimentation

during centrifugation. The purified MXene dispersion was concentrated by additional centrifugation, sealed with parafilm, and stored at approximately 5 °C for future use.

S3. Synthesis of MXene/Gelatin (MG) solution

To prepare the gelatin base solution, 0.2 g of gelatin powder was added to 10 mL of deionized water and stirred until completely dissolved and transparent at 40°C. Meanwhile, the concentrated MXene aqueous dispersion solution was prepared using the previously described method and adjusted to the desired concentration (28 mg mL^{-1}). The gelatin solution was added to the MXene dispersion solution at a volume ratio of 10% and gently stirred until uniformly mixed. The resulting MG composite solution was maintained at 40°C before further processing to prevent premature gelation.

S4. Fabrication of MG non-woven fabrics

The MG composite solution was stored at 40°C, then loaded into a syringe equipped with a 22G, 24G and 27G metal needle according to the target fiber diameter, and extruded into a 0.2 M NH_4Cl aqueous solution coagulation bath at room temperature by the wet spinning method. Upon contact with the ionic medium, the MG solution rapidly solidified into fibers through the physical gelation process of the gelatin component. The fibers were continuously collected, washed with DI water, and then rewashed with 0.1 M HCl to remove excess salt. The spun fibers were transferred to a porous membrane filter and assembled into a layered nonwoven mat through vacuum-assisted filtering. At this stage, the vacuum pressure ensured close intertwining of the fibers and uniform distribution, guaranteeing a cohesive fabric-like network with uniform density. The formed MG nonwoven fabric was dried overnight at room temperature under natural conditions before further use for biosignal monitoring applications.

S5. Characterization

Scanning electron microscopy (SEM) images of the cross-section and surface of membranes were obtained using a Hitachi S-4700 microscope. X-ray photoelectron spectroscopy (XPS) was performed using a theta probe base system (Al-Kα radiation; Thermo Fisher Scientific Inc.) equipped with an electron flood gun. X-ray diffraction (XRD) analyses were performed using a Rigaku MiniFlex benchtop X-ray diffractometer with a Cu-Kα radiation source (λ = 1.5418 Å). Fourier-transform Infrared spectroscopy (FT-IR) was performed using Bruker Optics Hyperion 1000 with Tensor 27. Atomic force microscopy (AFM) was performed using Bruker nanoIR3. Electrochemical impedance spectroscopy was performed over a frequency range from 1 Hz to 100 kHz. Equivalent circuits were used to fit the data and analyze the circuit components. In addition, we evaluated the product in wet and dry conditions for comparison with actual wear. To evaluate mechanical durability and wear resistance, a rheometer-based wear test was performed using PDMS elastomer counterfaces in a parallel plate configuration. Periodic friction was applied at a controlled normal force (0.1 N) and a frequency of 1 Hz for up to 70 cycles, followed by measurement of electrical resistance. After the test, SEM imaging was used to confirm fiber structure.

S6. Preparation of skin replica

To fabricate a skin-mimicking replica, a two-step molding process was used with Dragon Skin™ and PDMS. A human skin surface (forearm) was selected as the master template. A 1:1 mixture of Dragon Skin™ Part A and Part B was prepared by weight and stirred thoroughly. The mixture was degassed under vacuum for 5–10 minutes to remove air bubbles, then gently poured over the skin surface. After curing at room temperature for ~30, the cured Dragon Skin mold was carefully peeled off, preserving fine surface topography. PDMS base and curing

agent were mixed at a 10:1 weight ratio and degassed. The PDMS mixture was poured into the Dragon Skin mold coated by ease release 200 and cured at 70 °C for 2 hours. Once fully crosslinked, the PDMS replica was demolded, resulting in a positive skin replica with microscale texture fidelity.

S7. Water permeability test

A simple evaporation method was used to evaluate the water vapor permeability of MG nonwoven fabrics and demonstrate the movement of moisture that may occur during use, such as sweat.[3] Briefly, 3 mL of deionized water was placed in a small glass container (3.5 cm of diameter), and the opening of the container was sealed with a filter membrane followed by the test fabric sample. The joints were securely fastened with rubber bands to prevent side leakage. The initial mass of the container was recorded, and the sample was stored in a humidity-controlled chamber maintained at body temperature (~38°C) and relative humidity (~45–50%). Using a precision balance (±0.1 mg), the mass decrease of the container filled with water was measured at regular intervals. The water vapor transmission rate (WVTR) was calculated by plotting the relationship between mass decrease and time and extracting the slope. For comparison, a general filter membrane was used as a control sample, and fabric samples made with different needles, i.e., having different densities, were used.

S8. Recycling process of MG non-woven fabric

To demonstrate the recyclability of MG nonwoven fibers, samples were immersed in deionized water at 40°C at a concentration of approximately 10 mg/mL. After gently stirring the dispersion, mild ultrasonic treatment was applied to promote the gelatin matrix's dissolution and the MXene plates' redispersion. The mixture was visually inspected after complete dissolution to confirm uniform dispersion and the absence of visible aggregates. The solution

was sequentially centrifuged to remove any undissolved particles formed during the process or large aggregates that may occur during use. The first centrifugation was performed at 2000 rpm for 10 minutes to remove large particles. The supernatant was subjected to three additional centrifugations at 8000 rpm for 10 minutes each to purify the dispersion further and concentrate the material. The purified MG dispersion was adjusted to a concentration suitable for fiber manufacturing. The recycled MG solution was reprocessed into new nonwoven fibers using the same wet spinning and vacuum-assisted filtering method as the original sample. The resulting recycled MG fibers maintained sufficient mechanical strength and electrical functionality for reuse in biosignal monitoring applications.

S9. Measurement of electrocardiograms (ECG) and electromyograms (EMG)

For electrophysiological monitoring, a healthy male volunteer participated in the process of recording. ECG recording was performed using a modified three-lead configuration. A MG fabric electrode was placed near the heart and two 3M™ Red Dot™ ECG electrodes were on the right upper chest, and near the navel to complete the electrode circuit. The electrodes were attached to the edges of non-woven fabric using transparent film dressing without conductive gel to ensure skin adhesion. Signal acquisition was recorded using a custom signal acquisition system based on the Analog Devices AD8232 analog front-end module. This module is designed explicitly for bioelectric signal acquisition. It features an integrated instrumentation amplifier and filtering circuits for common-mode noise rejection, making it suitable for low-amplitude bioelectric signals (e.g., electrocardiograms). The AD8232 module incorporates on-chip high-frequency filtering (~0.5 Hz) to suppress baseline drift and low-frequency filtering (~40–100 Hz) to remove high-frequency noise and muscle artifacts. The module's analog output is digitized via a microcontroller (SparkFun RedBoards programmed with Arduino) and sampled at 500 Hz. The signal is streamed to a PC and visualized in real-time using custom

software or serial plotting tools. This configuration enabled stable detection of characteristic ECG signals such as P, QRS, and T waves under resting and mild exercise conditions while maintaining sufficient signal-to-noise ratio (SNR). Signal quality was evaluated before and after the reuse of the MG fabric electrodes to confirm performance maintenance.

EMG measurements were performed by placing MG electrodes on the biceps muscle. One MG electrode was placed on the middle part of the muscle, the 3M™ Red Dot™ ECG monitoring electrodes was placed proximally near the upper part of the upper arm, and on the bony area near the elbow. The subject was asked to perform repetitive voluntary flexion contractions of the biceps muscle. EMG signals were acquired using the Myoware 2.0 Muscle Sensor Module (Advancer Technologies). This module is a compact and user-friendly analog front end designed for surface EMG applications, featuring built-in amplification, rectification, and envelope detection and providing simultaneous multi-channel output. All signals were sampled at 1000 Hz using a microcontroller and recorded for post-processing. Participants performed voluntary isometric contractions at ~3-second intervals. EMG signal quality was evaluated based on waveform clarity and root mean square (RMS) amplitude, enabling quantitative comparisons between fabric densities and original and recycled MG electrodes.

To evaluate long-term usability and sweat resistance, ECG signals were recorded for 10 hours per day (total of 10 hours) for five consecutive days using the same fabric-based electrodes. The electrodes were reused between sessions without washing. For long-term EMG monitoring, the same electrodes used for ECG were used for five days to evaluate signal stability over time following ECG measurements. To simulate sweat exposure, the test was repeated under artificial sweat conditions using a standard artificial sweat solution.

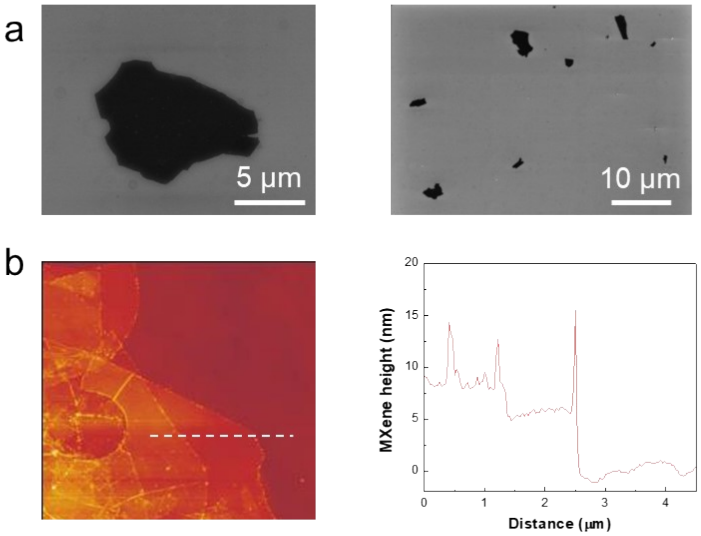

Figure S1. SEM images of MXene single sheets. (b) AFM image of MXene sheets and their height profile.

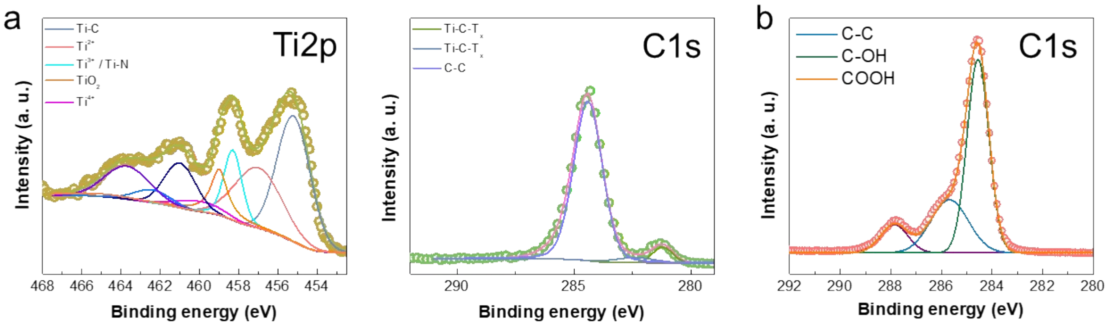

Figure S2. (a) Cross-sectional SEM image of pristine MXene. (b) Surface SEM image of MX–OAm–PLA membrane.

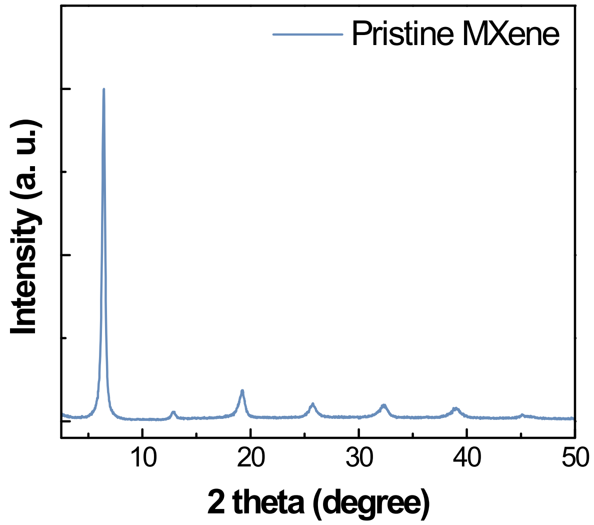

Figure S3. XRD spectra of pristine MXene.

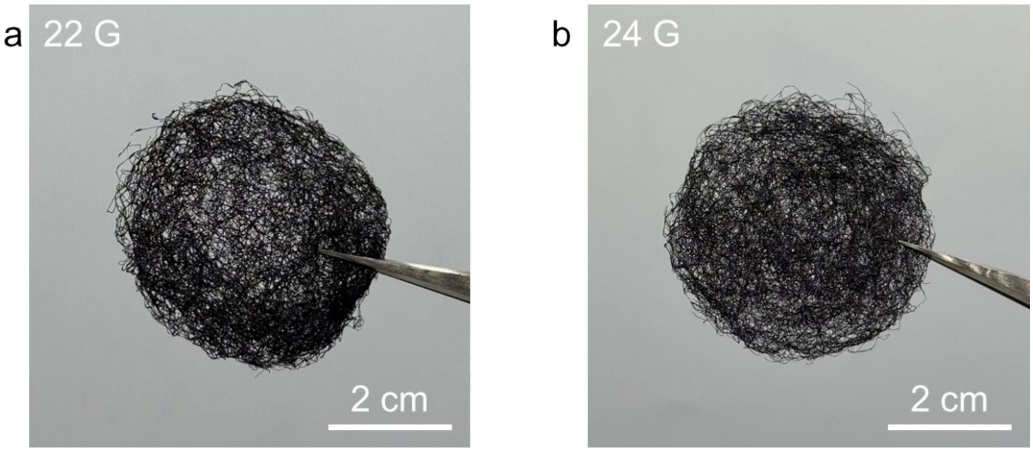

Figure S4. Photographic images of MG non-woven fabrics fabricated using (a) 22G (Total solution volume: 2.5 mL) and (b) 24G (Total solution volume: 2 mL) needles. The fabrics derived from the 22G and 24G show coarser fibers with reduced inter-fiber contact, resulting in lower out-of-plane conductivity despite higher spinning volumes.

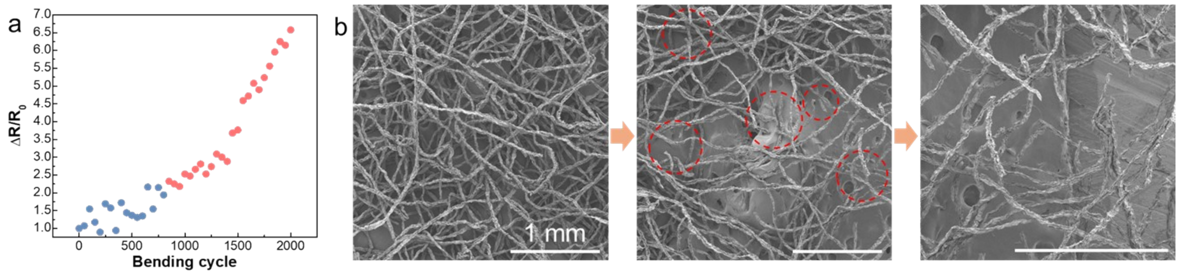

Fig. S5. Fatigue behavior and crack evolution of non-woven fabric under repeated bending. (a) Resistance changes of the fabric measured for bending cycles exceeding 2000 repetitions. (b) SEM images showing the progression of crack formation within the fabric structure as a function of bending cycles.

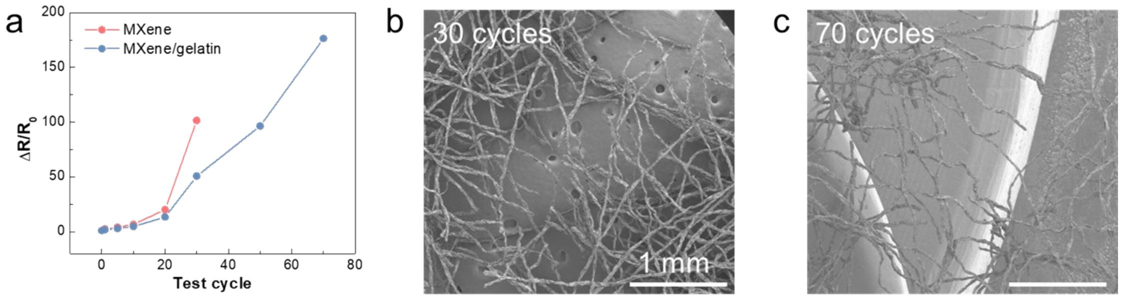

Fig. S6. Wear test results of MXene and MXene/gelatin samples. (a) Resistance changes of MXene and MXene/gelatin samples as a function of wear cycles. (b) SEM images showing progressive structural degradation in the MXene/gelatin sample over time. The left image shows the initial stage with minimal cracking, while the right image shows advanced crack formation and increased fiber detachment.

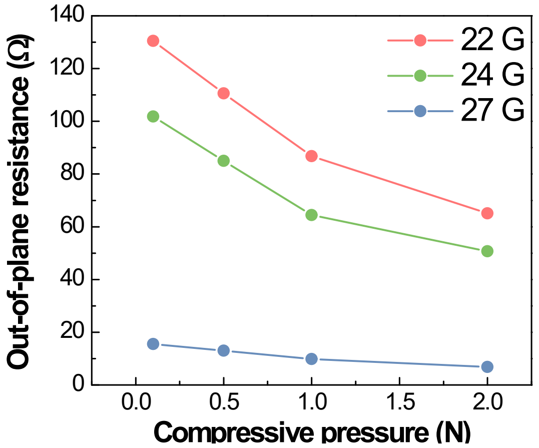

Fig. S7. Out-of-plane electrical resistance of non-woven fabrics produced with 22G, 24G, and 27G needle-spun fibers, measured under applied normal forces of 0.1, 0.5, 1, and 2 N.

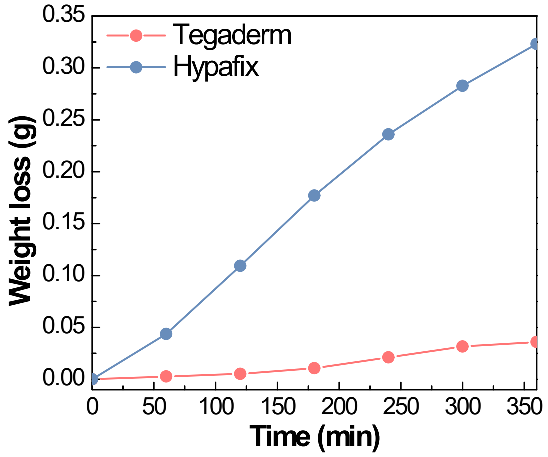

Fig. S8. Water vapor transmission rates of commercial patches.

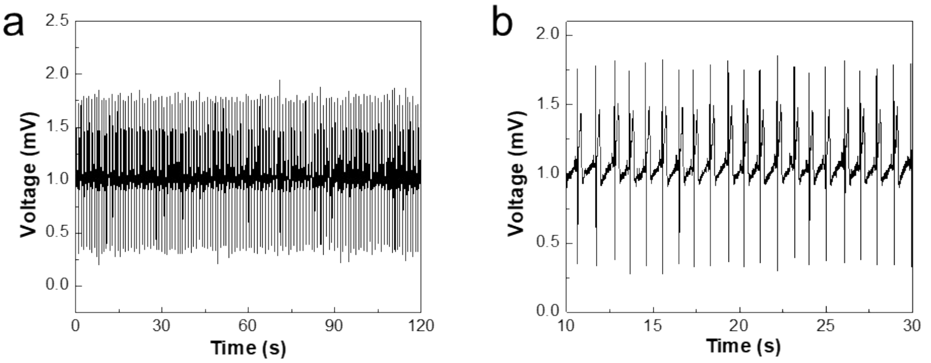

Fig. S9. Effects of sweat exposure on ECG signal. (a) ECG signal recorded for a 2-minute period under sweat exposure. (b) Enlarged view of the selected ECG segment to highlight signal characteristics affected by sweat.

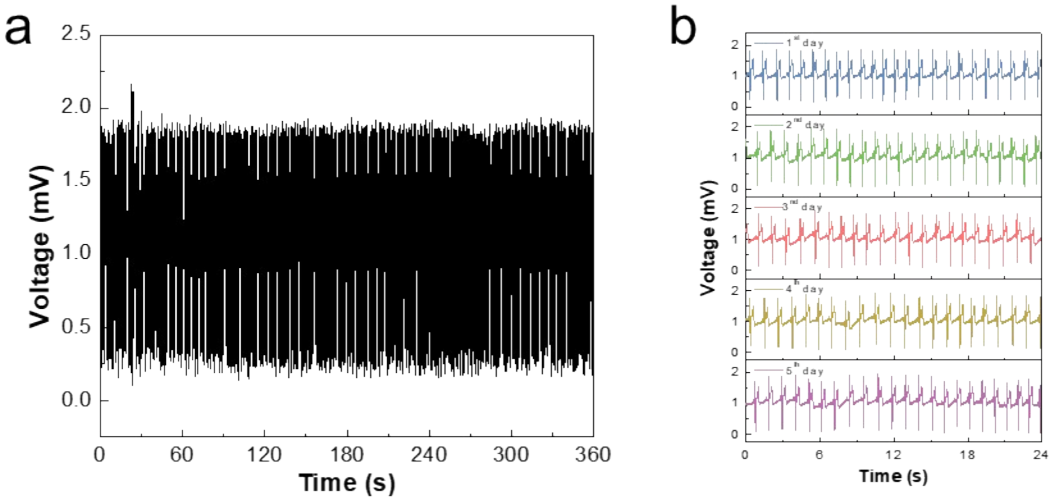

Fig. S10. Long-term ECG measurement. (a) ECG signal recording over a continuous 4-minute period. (b) ECG measurements conducted over five consecutive days, with 2-hour recordings each day.

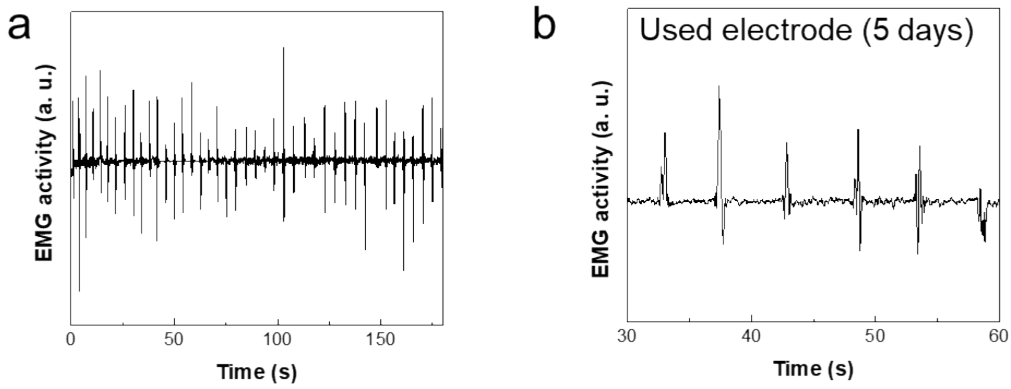

Fig. S11. EMG measurement and electrode durability. (a) EMG signal recorded for a 3-minute period. (b) EMG signal measured using the same electrode after 5 days of use, demonstrating maintained signal quality and electrode performance over time.

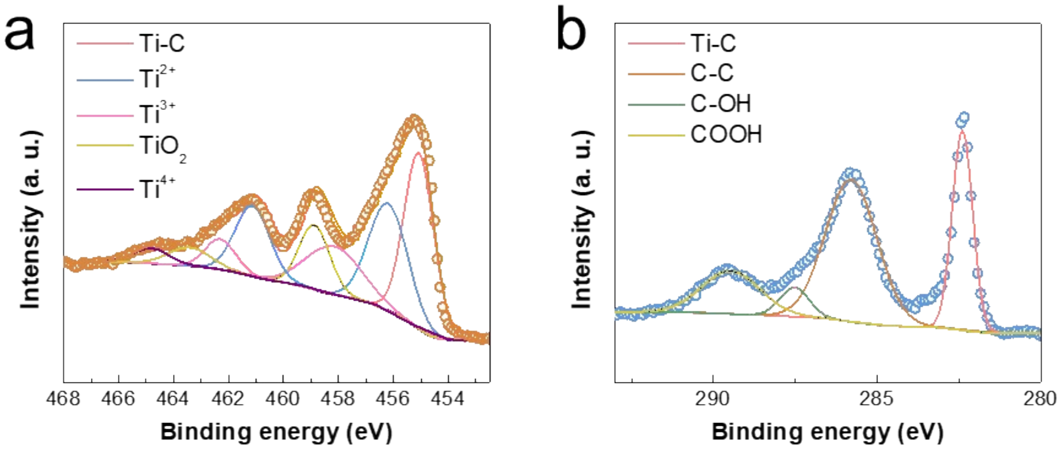

Fig. S12. XPS analysis of the fabric after three recycling cycles. (a) Ti 2p and (b) C 1s spectra of the chemical states of the fabric surface after the third recycle.

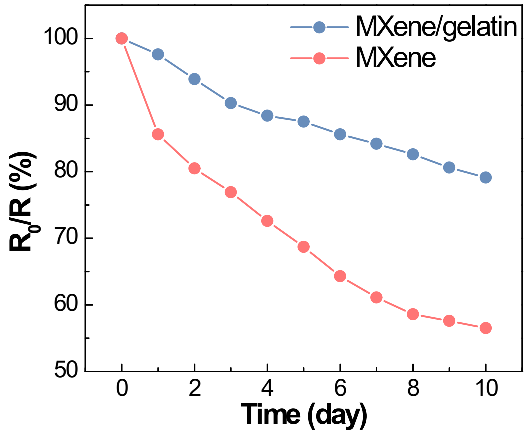

Fig. S13. Real-time resistance monitoring under high humidity (100% RH). Resistance changes of MXene and MXene/gelatin fabrics monitored for 10 days while stored under 100% relative humidity conditions.

REFERENCES

1. W. Eom, H. Shin, R. B. Ambade, S. H. Lee, K. H. Lee, D. J. Kang, T. H. Han, Large-scale wet-spinning of highly electroconductive MXene fibers. *Nat. Commun.*, 2020, *11*, 2825.

2. H. Shin, W. Eom, K. H. Lee, W. Jeong, D. J. Kang, T. H. Han, Highly electroconductive and mechanically strong Ti3C2T x MXene fibers using a deformable MXene gel. *ACS Nano*, 2021, *15*, 3320-3329.

3. L. Ding, C. Hang, S. Yang, J. Qi, R. Dong, Y. Zhang, H. Sun, Y. Jiang, X. In situ deposition of skin-adhesive liquid metal particles with robust wear resistance for epidermal electronics. *Nano Lett.*, 2022, *22*, 4482-4490.
